# 4Pipe4 – A 454 data analysis pipeline for SNP detection in datasets with no reference sequence or strain information

**DOI:** 10.1186/s12859-016-0892-1

**Published:** 2016-01-19

**Authors:** Francisco Pina-Martins, Bruno M. Vieira, Sofia G. Seabra, Dora Batista, Octávio S. Paulo

**Affiliations:** Departamento de Biologia Animal, Faculdade de Ciências, Computational Biology and Population Genomics Group, cE3c - Centre for Ecology, Evolution and Environmental Changes, Universidade de Lisboa, Campo Grande, 1749-016 Lisboa, Portugal; Departamento de Biologia e CESAM, Univ. de Aveiro, Aveiro, Portugal; Wurm Lab, School of Biological & Chemical Sciences, Queen Mary University of London, Mile End Road, E1 4NS London, UK; Centro de Investigação das Ferrugens do Cafeeiro (CIFC), Instituto Superior de Agronomia (ISA), Universidade de Lisboa, Quinta do Marquês, 2784-505 Oeiras, Portugal

**Keywords:** Genomics, SNP detection, Next generation sequencing, 454, Pipeline, EST, Non model organisms

## Abstract

**Background:**

Next-generation sequencing datasets are becoming more frequent, and their use in population studies is becoming widespread. For non-model species, without a reference genome, it is possible from a panel of individuals to identify a set of SNPs that can be used for further population genotyping. However the lack of a reference genome to which the sequenced data could be compared makes the finding of SNPs more troublesome. Additionally when the data sources (strains) are not identified (e.g. in datasets of pooled individuals), the problem of finding reliable variation in these datasets can become much more difficult due to the lack of specialized software for this specific task.

**Results:**

Here we describe 4Pipe4, a 454 data analysis pipeline particularly focused on SNP detection when no reference or strain information is available. It uses a command line interface to automatically call other programs, parse their outputs and summarize the results. The variation detection routine is built-in in the program itself. Despite being optimized for SNP mining in 454 EST data, it is flexible enough to automate the analysis of genomic data or even data from other NGS technologies. 4Pipe4 will output several HTML formatted reports with metrics on many of the most common assembly values, as well as on all the variation found. There is also a module available for finding putative SSRs in the analysed datasets.

**Conclusions:**

This program can be especially useful for researchers that have 454 datasets of a panel of pooled individuals and want to discover and characterize SNPs for subsequent individual genotyping with customized genotyping arrays. In comparison with other SNP detection approaches, 4Pipe4 showed the best validation ratio, retrieving a smaller number of SNPs but with a considerably lower false positive rate than other methods.

4Pipe4’s source code is available at https://github.com/StuntsPT/4Pipe4.

## Background

With the democratization of NGS technologies, large amounts of genomic and transcriptomic data became available to scientists in a short time span [1]. However, this magnitude of sequence data has brought most researchers a new bioinformatics challenge: to analyse and mine very large datasets [2]. One of the areas of particular interest of NGS data analysis is the detection of sequence polymorphisms. This task, however, becomes particularly difficult when no reference genome is available, which is common in non model organisms. This problem is somewhat mitigated when the samples can be accurately identified (strain information is present) [3]. However, if neither of these is accessible – such as in datasets with pools of individuals, looking for reliable variation can be a real problem. It was for this purpose that 4Pipe4 was developed: to find variation in 454 EST datasets where no reference sequence or strain information is available. This is especially useful for researchers who wish to find reliable variation in a panel dataset of pooled individuals to use as a starting point for designing genotyping arrays to further explore their data. The pipeline can provide very high quality SNPs as well as the flanking region sequence, necessary for the design of customized genotyping arrays, currently the most efficient way to extend SNP genotyping from those found in a panel of samples to a larger set of individuals for population genomic studies [4, 5].

Due to the nature of NGS data, any automated pipeline has to be strict enough as to follow a work-flow but, at the same time, flexible enough to serve the different purposes of each investigator. This is the role that 4Pipe4 intends to take. Although 4Pipe4 is tuned for EST data, it can also be used with genomic data and, to some extent, to help automate the process of gene discovery.

## Implementation

4Pipe4 is written in Python 3 and is licensed under the GPLv3. It is written in a modular manner that allows for relatively simple expansion of functionality.

Most of the functions present in 4Pipe4 result from the automation of already existing programs and the integration of their respective outputs. However, the variation detection routines are of original design and are based on three criteria, all of which can be adjusted by the user:

- *Base coverage –* The minimum required coverage (C); the default value is 15;
- *Base variants –* The minimum number of equal base variants required in a position (vmin); the default value is 20 % of the minimum required coverage;
- *Base quality –* The average minimum quality of each of the base variants (Qmin); the default value is 70.

This means that in order to consider a position of the alignment as a putative SNP, the below condition must be verified:

$$ \sum R\kern0.3em \ge \kern0.3em C\wedge \sum \mathrm{V}2\kern0.3em \ge \kern0.5em {v}_{\min}\wedge \overline{Q_{\mathrm{V}1}}\ge \kern0.3em {Q}_{\min}\wedge \overline{Q_{\mathrm{V}2}}\ge \kern0.3em {Q}_{\min } $$

Where “R” is the number of reads in the considered position, “C” is the minimum coverage as defined by the user, “V1” is the most frequent variant base type in the considered position, “V2” is the second most frequent variant base type in the considered position and “Q” is the quality value.

4Pipe4 uses a configuration file, called “4Pipe4rc” with a simple and self documented syntax for setting variables such as the location of programs, the SNP detection criteria and the parameters that should be passed to the external software. How the program uses this configuration file is explained in detail in the program documentation.

The analysis process is divided in 9 steps, each of which can be excluded from the run by issuing the appropriate arguments at run time. In step 1, 4Pipe4 takes an SFF file and, if all the steps are run, step 9 outputs a series of HTML formatted reports, compressed in *7zip*. Steps 7 (Gene Ontology) and 8 (SSR detection) are considered optional since they are not required for the SNP detection routines. 4Pipe4 requires the use of external programs, which can all be installed locally without root privileges (except Blast2GO which requires a MySQL database). The distribution comes with a set of helper scripts to automatically download and install all of the required software. All of the required programs are available under open-source licenses and are free to use (except Blast2GO which is not open source, but is free to use).

## Results and Discussion

### The analysis process

**Fig. 1:**
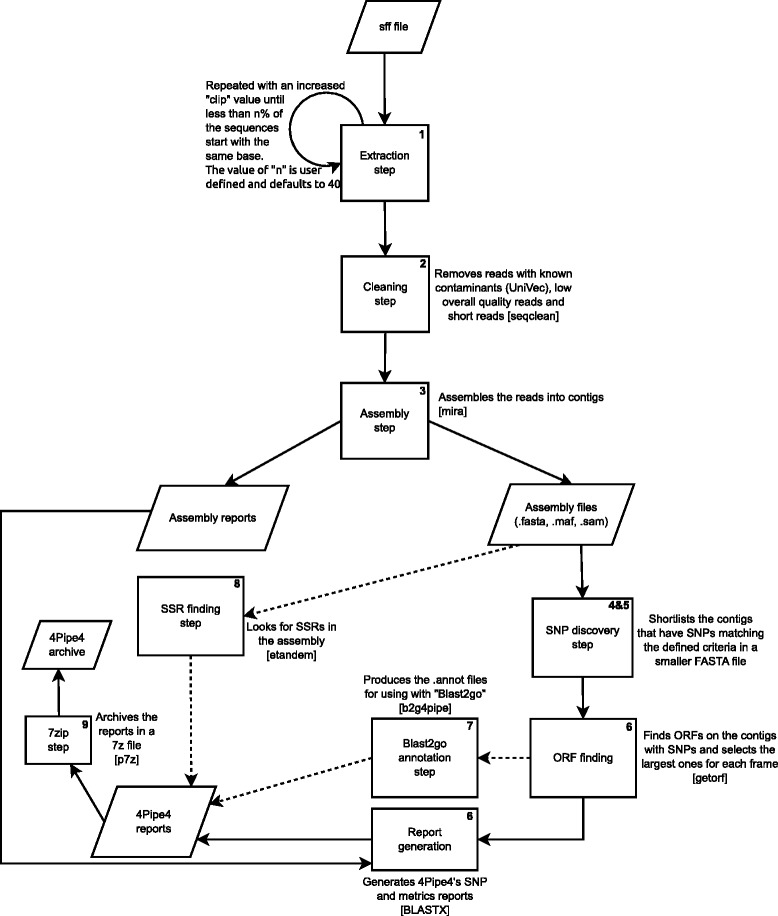
4Pipe4 flowchart. The rectangular shapes represent processes, the rhomboid shapes represent input/output files. The dashed arrows represent optional steps. The names inside square brackets are the names of the used external programs. The digits on the top right corner of each rectangle represent the step number of each process

The above mentioned 9 steps can be described as follows (See Fig. 1 for a more graphical overview):

- Step 1 – Extraction of the “FASTA” and “FASTA.QUAL” files from the original “SFF” file. This step can be skipped if not dealing with 454 data.
- Step 2 – “Cleaning” the sequences, by discarding low overall quality and short reads, as well as reads that contain contaminants matched against the “UNIVEC” database [6] or any other contaminant database at the user’s discretion. This step uses the “Sequence Cleaner” program [7] and can also be skipped if dealing with Illumina data.
- Step 3 – Assembling. This step uses mira [8]. A set of optimized parameters for SNP calling is contained in the example configuration file.
- Steps 4 and 5 – SNP gathering. Resorting to the “MAF” output from step 3 (which is converted into the “SAM” [9] format), potential SNPs are identified in the assembly. The result is a summary intermediate “TCS” file and a “FASTA” file including all the “contigs” that contain putative SNPs (which are identified in the sequence title). The software “pysam” [9, 10] is used in this step.
- Step 6 – Characterization of the detected SNPs, by attempting to fit them into Open Reading Frames (ORFs). The result is a “FASTA” file containing the ORFs with the SNPs identified in the sequence title, as well as the ORF frame allowing the quick assessment of the length and level of conservation of the SNP’s flanking region. This step uses the “EMBOSS getorf” program [11]. Also in this step, BLASTx [12] is run with the resulting ORFs against a large protein database, such as NCBI’s “nr”. Lastly, this step will produce an HTML formatted report with the characterized SNPs for easy referencing. The report is formatted as a table and can easily be transferred to any spreadsheet software for further data exploring. Another output of this step is an additional HTML report with a compilation of various dataset metrics.
- Step 7 (optional) – Blast2GO annotation; this step queries the contigs that contain SNPs against a large protein database such as NCBI’s ‘nr’ using BLASTx; these are then run through Blast2GO [13] using Blast2Go4Pipe, resulting in an annotation file that can be further analysed with Blast2GO itself.
- Step 8 (optional) – SSR detection, by using “EMBOSS etandem” to detect potential SSRs in the assembly. The required quality of the putative SSRs is defined in the configuration file.
- Step 9 – Compression of all the relevant result files into a 7zip archive which simplifies the transfer of (often large) results.

### Example usage

A test dataset with documentation on example usage is provided with the software package. An example resulting report is also provided for the test dataset (run with default values on all settings).

### Validation

In order to assess the efficiency of SNP detection and the rate of false positives, and assess the best default values to use, an approach using reference data was used.

For this goal, two reference sequences of two *E. coli* strains were used (http://www.ncbi.nlm.nih.gov/Traces/wgs/?val=ADWQ01 - Strain MS 85 and http://www.ncbi.nlm.nih.gov/Traces/wgs/?val=ADWR01 - Strain MS 79). Two 454 datasets were also downloaded from the NCBI Sequence Read Archive (SRA) [14] (http://www.ncbi.nlm.nih.gov/sra/SRX036805 and http://www.ncbi.nlm.nih.gov/sra/SRX036804) for the same strains as the references.

To assess the number of SNPs between the strains that could be found on the 454 datasets, the 454 reads of one strain were mapped against the reference sequences of the other strain using bowtie2 [15]. Atlas-SNP2 [16] reported 29673 SNPs between the reference sequence ′85′ and the 454 reads of the strain ′79′, and 28525 SNPs between the reference sequence ′79′ and the 454 reads of the strain ′85′.

4Pipe4 was then run on the two merged 454 datasets, discarding all strain information.

Although this validation method is not as good as true wet-lab genotyping, it is likely to be a good proxy, since Atlas-SNP2 is known to have very high sensitivity and specificity when dealing with 454 datasets [16].

The results varied with the different tested parameters (Table 1), but the best output was obtained with the default values of minimum coverage of 15 and minimum average quality of 70 per variant. This setup retrieved 114 SNPs, of which 32 did not match to any of those detected by Atlas-SNP2, being thus, considered false positives (28.07 % false discovery rate).

**Table 1:** Obtained and validated SNPs per parameter set. Of the six tested parameter combinations, the lowest false positive rate was retrieved with the default values: 15 Minimum coverage and 70 Minimum average quality

	Parameters used (Min. Average Quality)					
	10|60	10|70	15|60	15|70 (Default)	10|75	20|70
Total SNPs retrieved	234	169	155	114	107	89
Confirmed SNPs	97	86	88	82	69	57
False Positive rate (%)	58.55	49.11	43.23	28.07	35.51	35.96

Although the number of provided SNPs is relatively low, due to the restrictive assembly and filtering parameters, we find this a good trade-off relative to the high confidence of the retrieved SNPs.

The task of SNP calling in 454 data has been performed before on organisms without a reference sequence or strain information, with varying degrees of false positives. One such study, conducted using custom scripts for SNP calling provided a false positive rate of 80 % on 4200 retrieved SNPs [17]. Another example, where the contigs of 283 SNPs were manually screened and selected, had a slightly better false positive rate of 45 % [18].

The above mentioned studies are not directly comparable to the results of the benchmark performed here, since they are performed on different datasets, nevertheless they can be used to infer that, in general, 4Pipe4 retrieves a smaller number of SNPs than other methods, but with a considerably lower false positive rate. Since the main goal of this pipeline is to provide the user with high confidence SNPs for genotyping arrays, a rate of 28.07 % false positives is a considerable improvement relative to the other mentioned approaches.

### 4Pipe4 compared to other software

Although 4Pipe4 is specifically designed for the purpose of detecting variation when no strain information or reference sequence is available, other software exists that can be used for the same purpose, but which differs from 4Pipe4 in some aspects:

QualitySNP [19] – Relies on CAP3 for clustering the reads (which is optimized for Sanger sequences, while 4Pipe4 uses mira, which is optimized for NGS data). Requires perl, PHP, a configured webserver and a MySQL database for SNP retrieval. This means that root access to the machine in which the program is being run on is required. Furthermore, QualitySNP has been superseded by the simpler and faster QualitySNPng [20].

AGSNP [21] – Relies on Newbler assembler for clustering, and if strain information is not available, it further requires combining 454 data with *Illumina* or *SOliD* data (4Pipe4 does not require multiple technologies data for SNP calling).

Still other programs exist for SNP detection, but they usually require either a reference sequence, such as Atlas-SNP2 or SAMtools, or strain information, such as discoSnp++ [22] (formerly kisSnp [3]) or DIAL [23].

There is, however, another program that can be used for the same purpose as 4Pipe4 – QualitySNPng. This program, however is not an analysis pipeline, but rather a SNP caller for read alignments. It has a graphical user interface, which can be disabled for use in servers, but still requires “Qt4” to be installed in the server (which is not frequent). In order to compare it with 4Pipe4, we have modified the program to be usable without “Qt4” installed (https://github.com/StuntsPT/QualitySNP) and provide a branch of 4Pipe4 which is ready to use QualitySNPng (https://github.com/StuntsPT/4Pipe4/tree/new_snp_caller), without requiring any further dependencies.

Benchmarking the results of 4Pipe4 with QualitySNPng as the SNP caller, more SNPs were returned (513|147 SNPs found with the default|tuned values) than with our SNP caller, but with a larger rate of false positives (only 60|22 SNPs were a match to those found by AtlasSNP2, meaning a false positive rate of 88.3 %|85 %). Therefore, the builtin SNP caller was kept as default, but QualitySNPng can still be used from its own git branch if desired.

For the sake of completeness, we also made the SNP calling on the benchmark dataset using the software discoSnp++ (which requires strain identification) with the most restrictive parameters, to minimize the number of false positives. This program retrieved 9226 SNPs, of which 5967 were considered true positives (false positive rate of 35.3 %). As expected, this method retrieves more SNPs than both 4Pipe4 and QualitySNPng since it takes advantage of strain information, but it still provides a somewhat higher false positive rate than 4Pipe4.

## Conclusions

We present here an automated analysis process specifically designed for SNP detection from 454 pyrosequencing transcriptome reads, which we named 4Pipe4. This is the first program specifically built to automate the whole process of finding putative SNPs in NGS datasets that lack both information regarding the origin of each read and a reference sequence. In-silico validation of 4Pipe4 results using previously analysed reference data revealed good performance in the calling of high confidence SNPs.

The 4Pipe4 pipeline, at the cost of retrieving a relatively low number of SNPs, has provided a lower rate of false positive SNPs than both an alternative SNP caller (QualitySNPng) and an alternative software that uses strain information (discoSnp++), as well as those obtained in previous studies that used different approaches for a similar type of data and goal.

Since the main purpose of this software is to retrieve high confidence SNPs for further exploring, we expect the incremental contributions it brings to improve, speed up and facilitate research on the field of population genomics.

Furthermore, we expect to implement new features in 4Pipe4, such as: graphics in the metrics report; indel variation finding; integration of alternative software (such as newbler for assembling instead of mira); process optimization for NGS technologies besides 454; switch from FASTA + FASTA.QUAL format to FASTQ. These are some of the planned features, but others can be requested and implemented, should there be demand for them.

## Availability and requirements

- **Project name:** 4Pipe4
- **Project home page:**https://github.com/StuntsPT/4Pipe4
- **Operating system(s):** Platform independent (some external programs may be linux specific)
- **Programming language:** Python
- **Other requirements:** Python 3.0 or higher for running the pipeline, several other programs for specific tasks (consult the README file)
- **License:** GNU GPLv3
- **Any restrictions to use by non-academics:** None
